# The Spatial Distribution and Distance-Based Regulatory Compliance of Private Medicine Outlets in Two Urban Districts of Dar es Salaam, Tanzania

**DOI:** 10.7759/cureus.100044

**Published:** 2025-12-24

**Authors:** Vicky P Manyanga, Ng'wisho Nyalali, Paul M Makoye, Raphael Z Sangeda, Nelson E Masota, Innocent J Daniel, Prosper Tibalinda, Eliangiringa Kaale

**Affiliations:** 1 Department of Medicinal Chemistry, Muhimbili University of Health and Allied Sciences, Dar es Salaam, TZA; 2 Pharmaceutical Research and Development Laboratory, Muhimbili University of Health and Allied Sciences, Dar es Salaam, TZA; 3 Department of Pharmaceutical Microbiology, Muhimbili University of Health and Allied Sciences, Dar es Salaam, TZA

**Keywords:** accredited drug dispensing outlets (addo), dar es salaam, geospatial analysis, pharmacy distribution, regulatory compliance, spatial access, tanzania

## Abstract

Background and objective

Pharmacies and Accredited Drug Dispensing Outlets (ADDOs) play a central role in expanding access to essential medicines in low- and middle-income countries. In Tanzania, national regulations mandate minimum separation distances to prevent excessive clustering and help ensure that all communities have reasonable access to essential medicines. However, the extent of compliance in rapidly urbanizing districts remains unclear. This study aimed to examine the spatial distribution of private medicine outlets (PMOs) in the Kinondoni and Ubungo districts of Dar es Salaam and assess adherence to regulatory distance requirements.

Methods

A cross-sectional geospatial survey was conducted across 18 urban centers. Outlet coordinates were collected through field enumeration using REDCap (Research Electronic Data Capture) mobile tools between July 14, 2019, and September 6, 2019. Official ward boundary shapefiles were obtained from the National Bureau of Statistics. Spatial patterns were analyzed by mapping PMO locations, estimating ward-level densities, calculating nearest-neighbor distances, and evaluating compliance with Tanzania's minimum-distance regulations of 150 m for same-type outlets and 500 m for ADDO-pharmacy pairs.

Results

A total of 377 PMOs were mapped, comprising 232 (61.5%) ADDOs and 145 (38.5%) pharmacies. The outlet distribution was highly uneven: some wards demonstrated dense clustering, while others showed sparse coverage. The median nearest-neighbor distance was 753 m, although distances ranged from <1.0 m to over 6.0 km. Overall, 110 (29.2%) of the outlets fell below the regulatory spacing thresholds. Compliance levels were similar across outlet types.

Conclusions

Marked clustering and substantial non-compliance with minimum distance regulations were observed in Kinondoni and Ubungo. While dense clustering may improve convenience in some neighborhoods, persistent gaps in other areas suggest emerging spatial inequities. Geospatially informed regulatory planning is required to enhance equitable access to essential medicines. The observed 29.2% non-compliance rate highlights a meaningful regulatory gap, indicating that existing minimum-distance rules are not consistently implemented. This has practical implications for market saturation, potential unfair competition, and uneven access across neighborhoods, reinforcing the need for targeted inspections and more proactive spatial oversight.

## Introduction

Access to essential medicines is a core component of universal health coverage; however, geographic accessibility remains highly variable across many low- and middle-income countries (LMICs) [1,2]. Studies have consistently shown that pharmacies and drug outlets tend to cluster in commercially active or better-resourced areas, leaving peripheral communities with fewer or sometimes no accessible medicine sources [3,4]. Similar patterns have been observed across other LMIC settings, where outlets concentrate along major roads and busy commercial corridors. This creates areas of high accessibility while leaving other neighborhoods relatively underserved. In many African and Asian settings, small private drug shops and pharmacies are critical access points for basic healthcare services, particularly where formal health facilities are unevenly distributed [5,6].

Tanzania has established the Accredited Drug Dispensing Outlet (ADDO) program to expand access to quality-assured medicines in underserved areas [7]. Although the program was initially designed for peri-urban and rural settings [8], ADDOs are now widespread in urban centers, raising concerns about clustering, regulatory oversight, and equitable access. National regulations define minimum separation distances between private medicine outlets (PMOs); however, compliance within complex urban environments remains poorly documented.

Geospatial analysis offers a powerful approach for assessing outlet distribution, identifying clustering, evaluating regulatory compliance, and highlighting geographic inequities [9]. Kinondoni and Ubungo, two densely urbanized districts in Dar es Salaam, provide a meaningful setting to study these patterns. This research aimed to (1) map the distribution of pharmacies and ADDOs, (2) quantify nearest-neighbor distances, and (3) assess compliance with national minimum-distance regulations. Unlike previous ADDO-focused studies that relied primarily on administrative records or small-area surveys, this work provides the first ward-level, Global Positioning System (GPS)-validated geospatial mapping of both pharmacies and ADDOs in Dar es Salaam, enabling a systematic assessment of distribution patterns and distance-based regulatory compliance.

## Materials and methods

Study setting

This study was conducted in Kinondoni and Ubungo, two large and rapidly urbanizing districts in Dar es Salaam, Tanzania. Historically, both districts had been administratively unified until 2015, when Ubungo was established as an independent district to accommodate population growth and improve local governance. The combined area represents one of the most commercially active zones in Dar es Salaam. It contains densely populated neighborhoods where PMOs serve as key access points for essential medicines. Kinondoni comprises 19 administrative wards, while Ubungo contains 12, forming the spatial framework for this investigation. 

Ward inclusion and exclusion

A total of 18 wards across the two districts were selected for inclusion. These wards were selected using a purposive, criterion-based sampling approach aligned with the study’s focus on densely populated and commercially active urban neighborhoods. Fourteen wards from Kinondoni, including Bunju, Hananasif, Kawe, Kunduchi, Kijitonyama, Kinondoni, Ndugumbi, Makumbusho, Mwananyamala, Tandale, Mikocheni, Magomeni, Mzimuni, and Msasani, and four from Ubungo, including Makurumla, Mburahati, Manzese, and Sinza, were surveyed. These wards were selected based on their population density, commercial concentration, and multiple PMOs, making them suitable for assessing spatial accessibility and regulatory compliance. Peripheral wards such as Mbezi Juu, Mabwepande, Mbweni, Wazo, and Makongo in Kinondoni, and Kibamba, Goba, Kwembe, Saranga, Kimara, Makuburi, and Mabibo in Ubungo, were excluded - primarily due to their distance from the urban center and their sparse distribution of outlets. These exclusions helped to maintain methodological consistency and avoid distortions caused by wards with very low densities or geographically isolated locations.

Identification of private medicine outlets

Private medicine outlets were identified using systematic street-level mapping. Trained mobile data collectors walked along all accessible streets and footpaths within the selected wards and recorded every identifiable PMO between July 14, 2019, and September 6, 2019. For each outlet, collectors captured the outlet name, outlet type (pharmacy or ADDO), ward, latitude and longitude coordinates, and a photograph of the storefront. Data were collected using the REDCap (Research Electronic Data Capture) mobile application deployed offline, allowing data entry even in areas without cellular connectivity. Once connectivity was restored, the data were synchronized with a secure REDCap server hosted at the Muhimbili University of Health and Allied Sciences (MUHAS).

Before full-scale data collection, a pilot validation was conducted using a subset of outlets to confirm the GPS accuracy and measurement consistency. The mean positional error was approximately ±5 meters, which was deemed adequate for urban geospatial analysis, and the final dataset was archived [10].

Data management and cleaning

After fieldwork, the data were downloaded from the REDCap server into a master database for cleaning and validation. Duplicate entries were removed, inconsistent ward names were corrected, and outlets with coordinates falling outside Kinondoni or Ubungo boundaries were excluded. The final cleaned dataset formed the basis for all spatial and compliance analyses. REDCap serves as the primary data capture and management system. REDCap is a secure web-based research platform that supports validated data entry, maintains audit trails, enables automated data export to analytical software, and provides structured tools for importing data from external sources [11,12].

Spatial data sources

Administrative boundary shapefiles for all wards were obtained from the National Bureau of Statistics (NBS) of Tanzania through the Census 2022 geodatabase. These shapefiles were generated during national census cartographic preparation and represent official administrative boundaries at the regional, district, ward, and street/village levels. All geospatial layers were harmonized to a single coordinate reference system to ensure accurate overlay with PMO coordinates. All spatial processing and distance calculations were conducted in R using the sf, dplyr, and ggplot2 packages under a harmonized WGS84 coordinate reference system (EPSG:4326).

Spatial analysis

Spatial analysis focused on the distribution and clustering of PMOs, ward-level density patterns, and nearest-neighbor distances. Coordinates were imported into a geospatial environment and overlaid on official ward polygons. Straight-line (Euclidean) distances were used to calculate the distance between each outlet and its closest neighboring outlet. This procedure follows the standard approaches used in previous pharmacy-mapping studies [4] and provides an objective measurement of spatial proximity. The analysis enabled the identification of clusters, spatial gaps, and marked variations in accessibility across wards.

Regulatory compliance assessment

Regulatory compliance was assessed according to Tanzania's minimum spacing requirements for PMOs. Pharmacies are required to maintain a minimum separation distance of 150 meters from other pharmacies, ADDOs must maintain at least 150 meters from other ADDOs, and ADDO-pharmacy pairs must maintain a separation of at least 500 meters, as stipulated in the national regulatory documents issued by the Ministry of Health [13,14]. For each outlet, the nearest-neighbor distance was compared with the applicable threshold and classified as either compliant or non-compliant.

Mapping and visualization

Mapping and visualization were performed using R statistical software with standard geospatial packages. The cleaned outlet coordinates were joined with official ward shapefiles obtained from the NBS, enabling an accurate overlay of point data on authoritative administrative boundaries. Four thematic map outputs were produced: a geospatial distribution map showing the locations of pharmacies and ADDOs, a compliance map indicating whether each outlet met the minimum-distance requirements, a ward-level density map using scaled bubble sizes to represent variation in outlet counts, and a nearest-neighbor distance heat map illustrating areas of clustering and dispersion. All maps were generated using consistent color schemes, clearly defined boundary lines, and a light-blue ocean background to enhance readability and support intuitive interpretation of spatial patterns.

Ethical approval

This study was approved by the Muhimbili University of Health and Allied Sciences Institutional Review Board, Reference: DA.282/298/01.C/MUHAS-REC-10-2020-379.

## Results

Distribution of private medicine outlets

Across the 18 surveyed wards in Kinondoni and Ubungo districts, 377 PMOs were mapped, comprising 232 ADDOs (61.5%) and 145 pharmacies (38.5%). Outlet numbers varied across wards, ranging from 83 in Bunju to 10-12 in Ndugumbi and Makumbusho, respectively. GPS coordinates were recorded with an accuracy of approximately ±5 meters (Table 1).

**Table 1:** Distribution of private medicine outlets by ward

Ward	N	Percent (%)
Bunju	83	22.0
Makurumla	41	10.9
Manzese	31	8.2
Hananasif	29	7.7
Mburahati	26	6.9
Kawe	24	6.4
Sinza	21	5.6
Kunduchi	18	4.8
Mikocheni	18	4.8
Kijitonyama	18	4.8
Mwananyamala	17	4.5
Kinondoni	17	4.5
Tandale	17	4.5
Mzimuni	17	4.5
Magomeni	13	3.4
Makumbusho	12	3.2
Ndugumbi	10	2.7
Msasani	8	2.1
Total	377	100.0

Distance characteristics

The shortest distances between outlets varied widely. Nearest-neighbor distances ranged from 0.40 m to 6,243.77 m, with a median of 753.06 m, a mean of 1,237.83 m, and an interquartile range of 379.20 - 1,939.17 m (Table 2).

**Table 2:** Descriptive statistics for nearest-neighbor distances (meters)

Statistic	Value
Number of outlets	377
Mean distance	1237.83
Median distance	753.06
Standard deviation	1361.78
Minimum	0.40
Maximum	6243.77
25th percentile	379.20
75th percentile	1939.17

Compliance with minimum-distance requirements

Of the 377 outlets recorded, 267 (70.8%) met the minimum distance requirement, whereas 110 (29.2%) did not. Both compliant and non-compliant outlets were observed across all the wards (Table 3).

**Table 3:** Private medicine outlets compliance summary

Compliant	N	Percent (%)
Yes	267	70.8
No	110	29.2
Total	377	100.0

Compliance by outlet type

Compliance did not vary substantially across the outlet categories. Among the ADDOs, 164 of 232 (70.7%) complied with spacing requirements, compared with 103 of 145 (71.0%) pharmacies. The overall compliance rate was 70.8% (Table 4).

**Table 4:** Compliance by outlet type

Outlet type	Compliant (N)	Non-compliant (N)	Total (N)	Compliance (%)
ADDO	164	68	232	70.7
Pharmacy	103	42	145	71.0
Total	267	110	377	70.8

Ward-level distance patterns

The ward-level distance summaries demonstrated wide numerical variability. Median nearest-neighbor distances ranged from 218.74 m in Bunju to 2,205.10 m in Hananasif. Maximum distances across wards extended from approximately 1.5 km to more than 6.0 km, indicating heterogeneous spatial patterns within the study area (Table 5).

**Table 5:** Ward-level nearest-neighbor distance statistics

Ward	N	Mean (m)	Median (m)	Min (m)	Max (m)
Bunju	82	712.08	218.74	0.53	6243.77
Hananasif	29	2174.16	2205.10	57.28	4215.92
Kawe	24	590.63	342.56	2.86	5874.53
Kijitonyama	18	1684.86	646.0	59.43	4455.27
Kinondoni	17	1582.06	742.63	15.08	4347.53
Kunduchi	18	576.92	330.98	3.25	3575.84
Magomeni	13	1292.78	463.17	20.92	3990.75
Makumbusho	12	1440.0	793.82	11.09	3871.85
Makurumla	41	1202.18	753.06	0.40	5213.60
Manzese	31	1323.97	820.40	18.36	5120.71
Mburahati	26	1427.02	1111.98	23.03	4122.61
Mikocheni	18	675.50	284.83	4.14	3531.17
Msasani	8	1090.42	521.48	2.55	3517.94
Mzimuni	17	1052.94	402.86	4.76	3949.19
Mwananyamala	17	882.50	439.67	1.42	4190.66
Ndugumbi	10	825.39	314.32	11.55	3898.68
Sinza	21	1301.40	838.11	9.18	4729.97
Tandale	17	1012.54	436.04	11.24	4215.47

Spatial mapping

**Figure 1:**
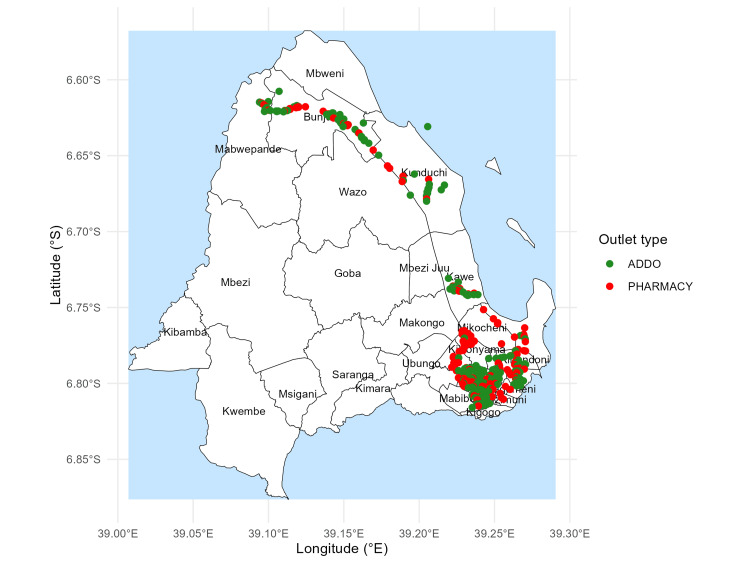
Geospatial distribution of pharmacies and ADDOs in Kinondoni and Ubungo This map illustrates the location of private medicine outlets across the two districts using official ward boundaries. Pharmacies and ADDOs are represented as point features overlaid on district polygons. Map created by the authors using R (ggplot2). Administrative boundaries are based on official ward shapefiles from the National Bureau of Statistics (NBS), Tanzania ADDOs: Accredited Drug Dispensing Outlets © OpenStreetMap contributors

Mapped locations of all 377 pharmacies and ADDOs across the 18 surveyed wards, overlaid on official ward boundaries, show tight clusters in wards such as Bunju, Kawe, Makurumla, and Mburahati (Figure 1).

**Figure 2:**
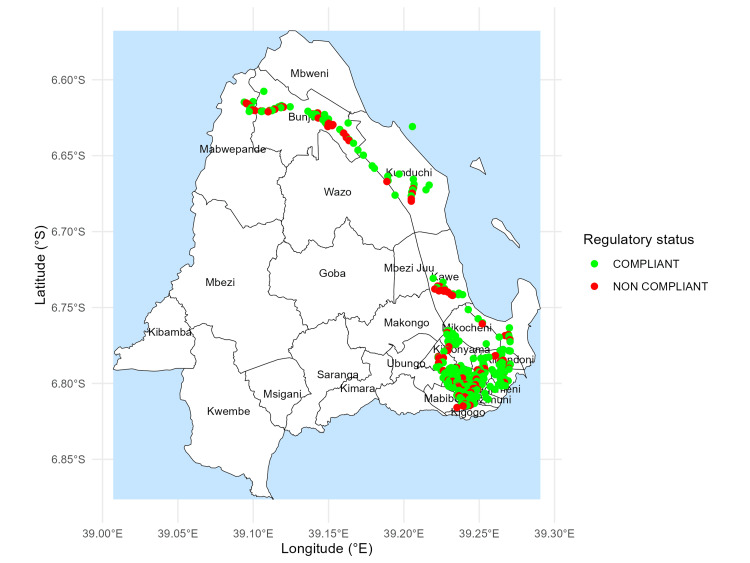
Compliance status of outlets based on minimum-distance regulations Outlets are classified as compliant or non-compliant according to the shortest distance to their nearest neighboring outlet, based on Tanzania’s regulatory spacing standards. Map created by the authors using R (ggplot2). Administrative boundaries are based on official ward shapefiles from the National Bureau of Statistics (NBS), Tanzania © OpenStreetMap contributors

Based on nearest-neighbour distances, both compliant and non-compliant outlets were identified in every surveyed ward, reflecting the application of the minimum-distance requirements across the study area (Figure 2).

**Figure 3:**
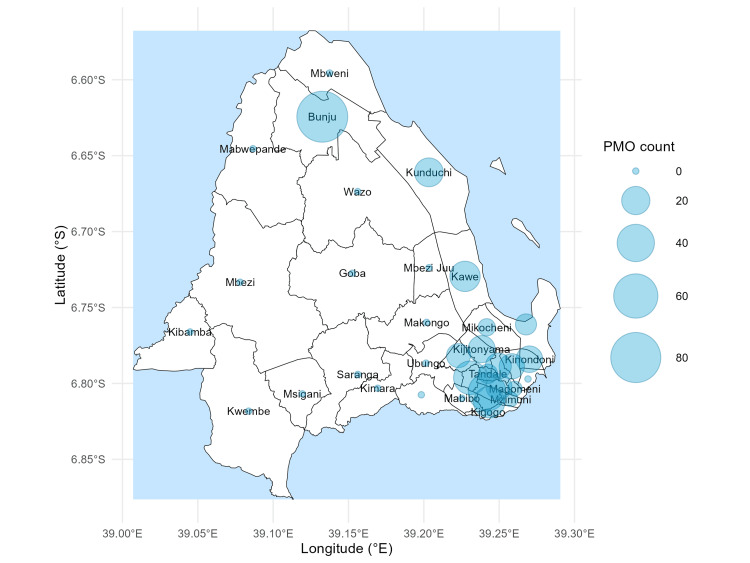
Ward-level density map of private medicine outlets Bubble sizes correspond to the number of outlets per ward, demonstrating variation in density across the study area. Surveyed and non-surveyed wards are distinguished by fill color. Map created by the authors using R (ggplot2). Administrative boundaries are based on official ward shapefiles from the National Bureau of Statistics (NBS), Tanzania © OpenStreetMap contributors

Bunju had the highest outlet density (83 PMOs), followed by Makurumla (41) and Manzese (31), while several wards had fewer than 15 outlets. These distributions are shown using scaled bubble sizes (Figure 3).

**Figure 4:**
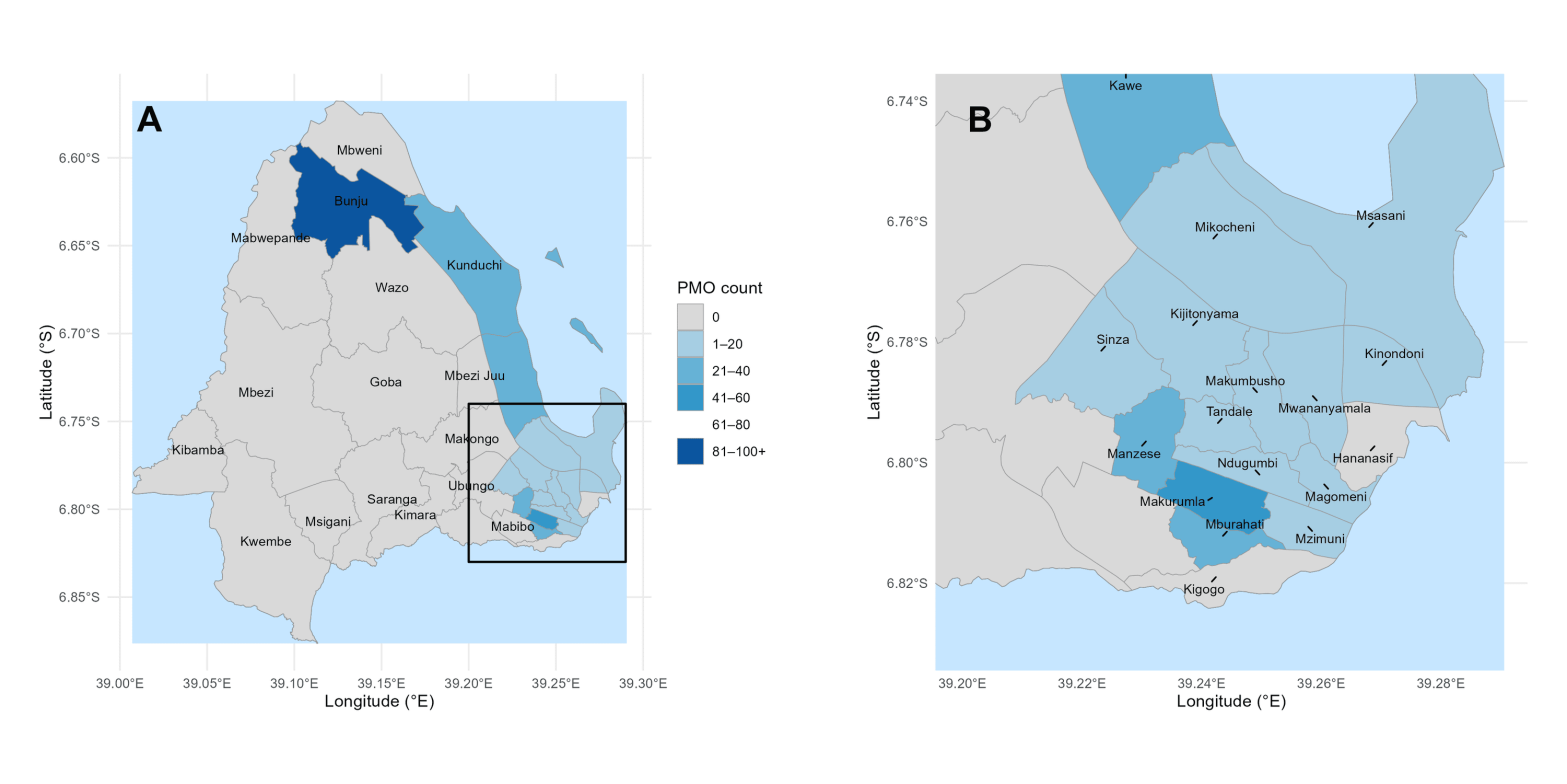
Nearest-neighbor distance heat map with zoomed southeastern cluster Panel A: Ward-level counts of private medicine outlets (PMOs) across Kinondoni and Ubungo districts. Darker shades indicate higher outlet density. A bounding box highlights the southeastern high-density cluster. Panel B: Zoomed-in view of the southeastern wards (Manzese, Makurumla, Mburahati, Ndugumbi, Magomeni, Kinondoni, Mwanyamala, Mzimuni, Hananasif, Kigogo), showing detailed distribution patterns All maps were created in R (ggplot2), using official ward shapefiles from the National Bureau of Statistics (NBS), Tanzania © OpenStreetMap contributors

Neighbourhood-level proximity patterns, illustrated in the heat map, confirm the clustering identified in Figure 1 and Figure 3, with the shortest distances concentrated in Kawe, Kunduchi, Mzimuni, and Bunju (Figure 4).

## Discussion

This study presents an updated geospatial profile of PMOs in the Kinondoni and Ubungo districts, revealing an uneven distribution, substantial clustering, and measurable non-compliance with minimum distance requirements. Similar to observations from other LMIC settings, outlets were concentrated along busy commercial corridors and major roads [6,9], creating areas of high accessibility while leaving other neighborhoods relatively underserved [3,4]. A notable finding was the predominance of ADDOs in these urban districts, despite regulatory provisions designating urban areas for pharmacy services [14]. This pattern is consistent with earlier reports of ADDOs encroaching into urban markets [8]. The presence of ADDOs in wards where they are not formally permitted may reflect gaps in enforcement, commercial incentives, or community demand for low-cost, nearby sources of medicines. Previous studies have documented similar tensions between formal regulatory expectations and the practical realities of medical provision in rapidly growing cities [15,16].

Proximity violations were common across both outlet categories. Nearly one-third (29%) of all PMOs operated within distances shorter than the regulatory thresholds. Comparable non-compliance has been observed in Bangladesh, India, and Myanmar, where market competition and population density often influence outlet placement more strongly than formal guidelines [5,6]. The similarity in compliance levels between pharmacies and ADDOs suggests that spacing violations arise from broader structural dynamics rather than behavior unique to one outlet type. Spatial disparities were also evident. While some wards had multiple outlets located within a few meters of one another, others had inter-outlet distances exceeding several kilometers. Such variation may contribute to inequities in timely access to essential medicines. Global evidence consistently shows that medicine outlets tend to cluster in areas of higher socioeconomic activity, with a reduced presence in lower-income or peripheral zones [4,17,18]. In settings facing similar challenges, telepharmacy and differentiated regulatory models have been proposed as mechanisms to improve access [18,19].

The strengths of this study include the use of validated GPS data, structured street-level mapping, and reliance on authoritative ward boundaries. The methodology enabled precise estimation of nearest-neighbor distances and clear identification of compliance gaps. The findings provide an evidence base to inform regulatory planning, highlight enforcement challenges, and support spatially guided decision-making. Future work could apply road network or travel time models, incorporate population density and service utilization metrics, and examine outlet dynamics longitudinally. Qualitative work involving outlet owners, community members, and regulators may also help clarify the drivers behind clustering, regulatory compliance patterns, and the persistence of ADDOs in urban areas.

In the context of Tanzania’s evolving pharmaceutical landscape, the observed clustering and spacing violations highlight structural pressures within densely populated urban districts. High commercial activity, limited regulatory visibility, and the persistence of ADDOs in zones where they are not formally authorized suggest that market forces may be outpacing regulatory oversight. These findings align with patterns reported in other sub-Saharan African settings, where retail pharmaceutical markets expand rapidly in urban cores while underserved peripheral areas remain relatively isolated. Integrating routine geospatial monitoring into regulatory workflows - supported by periodic field validation - could help institutions identify compliance hotspots earlier and allocate inspection resources more efficiently.

Limitations

This study has several limitations. First, the selection of surveyed wards focused on high-density areas. It may not fully represent the broader geospatial distribution of outlets across all wards in the Dar es Salaam region and Tanzania. Peripheral wards with dispersed settlement structures were excluded in the Ubungo district, potentially underestimating the extent of spatial inequity. These excluded peripheral wards comprise a substantial proportion of the total geographic area of both districts, which may limit the generalizability of the findings to more sparsely populated or peri-urban settings. Second, Euclidean distances were used rather than road-network-based distances, which may not accurately reflect actual travel times or accessibility barriers. Third, regulatory exceptions, such as those related to highways, population density, or discretionary approvals, could not be incorporated into the compliance assessment.

Finally, the study did not integrate demographic or socioeconomic data, which limits its ability to interpret whether outlet distribution aligns with population needs. In addition, because the study did not include socioeconomic or environmental covariates, it was not possible to examine how contextual factors may influence or explain the observed spatial patterns of PMO distribution. Finally, the survey reflects a single time-point snapshot and therefore does not capture potential seasonal or temporal variations in outlet activity. During fieldwork, the operational status of outlets could not be independently verified, as the focus was on locating and geo-referencing outlets rather than conducting regulatory inspections. These temporal considerations should be taken into account when interpreting the stability of outlet presence across wards.

## Conclusions

This geospatial assessment revealed significant clustering, uneven distribution, and widespread spacing violations among pharmacies and ADDOs in Kinondoni and Ubungo. Although these are urban districts where pharmacies would typically be expected to dominate, ADDOs were more prevalent and were found in several wards where they are not permitted. Nearly one-third of all outlets failed to meet minimum-distance regulations. This level of non-compliance has practical implications, including potential market saturation in already-dense areas, uneven service coverage, and regulatory challenges in ensuring fair and equitable access, indicating the need for strengthened oversight, more explicit operational guidance, and improved enforcement mechanisms. Persistent spatial disparities suggest that some communities may have reduced access to essential medicines. Incorporating geospatial intelligence into regulatory planning, along with road network analysis, population data, and qualitative insights from stakeholders, may support a more equitable and efficient PMO distribution strategy as Dar es Salaam continues to expand.
